# Identification of QTL for Stem Traits in Wheat (*Triticum aestivum* L.)

**DOI:** 10.3389/fpls.2022.962253

**Published:** 2022-07-14

**Authors:** Yanan Niu, Tianxiao Chen, Chenchen Zhao, Ce Guo, Meixue Zhou

**Affiliations:** ^1^Tasmanian Institute of Agriculture, University of Tasmania, Hobart, TAS, Australia; ^2^College of Agronomy, Shanxi Agricultural University, Taigu, China

**Keywords:** lodging, genetic map, vascular bundle characters, plant height, yield contributing traits

## Abstract

Lodging in wheat (*Triticum aestivum* L.) is a complicated phenomenon that is influenced by physiological, genetics, and external factors. It causes a great yield loss and reduces grain quality and mechanical harvesting efficiency. Lodging resistance is contributed by various traits, including increased stem strength. The aim of this study was to map quantitative trait loci (QTL) controlling stem strength-related features (the number of big vascular bundles, stem diameter, stem wall thickness) using a doubled haploid (DH) population derived from a cross between Baiqimai and Neixiang 5. Field experiments were conducted during 2020–2022, and glasshouse experiments were conducted during 2021–2022. Significant genetic variations were observed for all measured traits, and they were all highly heritable. Fifteen QTL for stem strength-related traits were identified on chromosomes 2D, 3A, 3B, 3D, 4B, 5A, 6B, 7A, and 7D, respectively, and 7 QTL for grain yield-related traits were identified on chromosomes 2B, 2D, 3D, 4B, 7A, and 7B, respectively. The superior allele of the major QTL for the number of big vascular bundle (VB) was independent of plant height (PH), making it possible to improve stem strength without a trade-off of PH, thus improving lodging resistance. VB also showed positive correlations with some of the yield components. The result will be useful for molecular marker-assisted selection (MAS) for high stem strength and high yield potential.

## Introduction

Lodging is defined as the permanent displacement of crop stems that can cause devastating agricultural losses such as significant reductions in crop yield and grain quality, as well as harvesting efficiency (Kokubo et al., 1989; Duan et al., 2004; Berry and Berry, 2015; Zhang et al., 2016a,b). Plant stem lodging is attributed to plant height, stem diameter and thickness, upper and lower internodal strength, stem wall thickness, lignin and cellulose accumulation within the stem wall, and spike weight (Zuber et al., 1999; Kong et al., 2013; Shah et al., 2019). Dwarf and semi-dwarf genes have been introduced into wheat breeding programs in recent years (Fischer and Stapper, 1987; Berry et al., 2004), however, positive effects on lodging resistance and grain yield have not always been observed for certain wheat dwarfing genes (Milach and Federizzi, 2001). In addition, 0.7 m of the minimum plant height for optimal grain yield has already been reached in certain genotypes in 1997 (Flintham et al., 1997), so shortening the height would not be the target for further improvement of crop lodging resistance. Furthermore, a recent study indicated that an increase of 28 N mm (the force required to bend a stem) in stem strength could reduce the probability of stem lodging from 0.73 to 0.59 of a crop 130 cm tall yielding 8.9 t ha^−1^ (Piñera-Chavez et al., 2021).

Wheat (*Triticum aestivum* L.) is the second most widely grown crop in the world. Its consumption is excepted to account for 19% of the calories in the global human diet (Aksoy and Beghin, 2004). With the vast improvement in wheat grain yield, the weight of stem and spike becomes the main loads for crop stems (Zuber et al., 1999; Kong et al., 2013). In cereals, stem diameter, culm wall thickness, and the number of big and small vascular bundles are related to stem breaking strength and bending strength against lodging (Kokubo et al., 1989; Duan et al., 2004; Wang et al., 2006; Packa et al., 2015). However, Kelbert et al. (2004) found no significant correlations among lodging resistance and other morphological characteristics such as stem diameter, stem wall thickness (Kelbert et al., 2004). The correlations between lodging resistance and the number and size of vascular bundles in cross-section of wheat stem have also been reported but results are inconsistent (Khanna, 1991; Zuber et al., 1999; Kelbert et al., 2004; Wang et al., 2006; Kong et al., 2013).

Many quantitative trait loci (QTL) in wheat have been reported for stem strength, culm wall thickness, pith diameter, and stem diameter (Hai et al., 2005; Berry and Berry, 2015). A single solid stem QTL identified on chromosome 3BL contributes to lodging resistance (Kong et al., 2013). From a wheat and spelt cross, a lodging resistance QTL was found to be related to plant height, culm stiffness, leaf width, leaf-growth, days to ear emergence, and culm thickness (Keller et al., 1999). Genes controlling culm diameter and wall thickness at the second basal internode contribute to lodging resistance with culm diameter displaying additive and partial dominant effects (Cui et al., 2002) and culm wall thickness showing both additive and nonadditive effects (Yong et al., 1998). A common genomic region affecting overall stem strength, which included internode material strength, internode diameter, and internode wall width, has been reported in the interval of 278–287 cM on chromosome 3B (Piñera-Chavez et al., 2021). Using 81 chromosome substitution lines between the red hard winter wheat cultivars, Wichita and Cheyenn, the Cheyenne chromosomes 2A and 4D had major effects the number of inner vascular bundles when substituted into Wichita (Al-Qaudhy et al., 1988). QTL for wheat vascular bundle system at 2 cm below the neck of the spike have been identified on chromosomes 1A, 2A, 2D, 5D, 6A, 6D, 7D (Sang et al., 2010).

The vascular bundle is an important structure of source-sink transport system which has a strong impact on the efficiency of photosynthetic production, mineral nutrients uptake, and water transportation (Housley and Peterson, 1982; Lucas et al., 2013). Significant positive correlations were observed between grain yield and the number of vascular bundles in rice (Mohammad et al., 2021), barley, oats (Housley and Peterson, 1982), and wheat (Evans et al., 1970). However, most studies have been focused on the vascular bundles within the upper segment of the plant, including the neck panicles (peduncle; Zhai et al., 2018; Fei et al., 2019), the rachis (Terao et al., 2010; Wolde and Schnurbusch, 2019) not within the stem, especially the basal stem (the third internode), which also contributes to lodging resistance (Duan et al., 2004). In Arabidopsis, genes involved in vascular bundle system, such as *MP*, *PHB*, *PHV*, *AtHB15*, and *REV*, have been identified (Hardtke and Berleth, 1998; McConnell et al., 2001; Zhong and Ye, 2004; Du and Wang, 2015). Genes affecting the vascular bundle system have also been reported in rice. Among them, *APO1* controls the number of primary rachis branches as well as the vascular bundle formation, *DEP1* regulates the number of larger vascular bundles and ABA signaling, and *NAL1* affects vein patterning and polar auxin transport (Qi et al., 2008; Terao et al., 2010; Fujita et al., 2013; Fei et al., 2019). *WHEAT ORTHOLOG OF APO1* (*WAPO1*) gene is an orthologue of the rice *APO1* gene, affecting spikelet number per spike, but no further evidence has been found for its function on the development of vascular bundle (Kuzay et al., 2019). Restricted genes such as *TmBr1* (Deng et al., 2019), an allele of the *Q* (Q*^c1^*) gene (Xu et al., 2018), are involved in the formation of vascular bundles that regulate the efficiency in transporting assimilates in the spikes or the stem strength against lodging in wheat. *TaCM*, involved in the biosynthesis of lignin, has also been implicated in stem strength (Ma, 2009). However, the functional genes have not yet been fully discovered, and the molecular mechanism of how the vascular bundle system influences crop yield is largely unknown.

Linkage mapping approaches based on individual and multiple populations have become routine in wheat genetic studies to dissect the genetic architecture of complex traits (Wang et al., 2014; Liu et al., 2020; Qu et al., 2021). QTL remains of great importance in identifying the genetic basis of the link between the anatomical and morphophysiological traits of the stem and lodging, and yield potential. In our present study, the number of big vascular bundle (VB), stem diameter (SD, mm), and stem wall thickness (SWT, mm) were examined in a doubled haploid (DH) population of 194 wheat lines under three environmental conditions. The objective of this study was to identify QTL for the vascular bundle system of the third internode along with other stem and panicle traits in wheat. Our results will assist in molecular marker assistant selection to obtain lines with high stem strength and grain-yielding capacity in wheat breeding.

## Materials and Methods

### Plant Materials

A double haploid (DH) population of 194 lines was generated by anther culture from the cross between two elite wheat cultivars “Baiqimai” (short and thin stem) and “Neixiang 5” (tall and thick stem). Field trials were conducted at Mt. Pleasant Laboratory in Tasmania, Australia (147°08’E, 41°280’S). Fifteen seeds of each line were sown on April 15, 2020 and April 25, 2021 in a row of 0.6 m with a row spacing of 0.3 m, following local farmers’ practices for field management. Under glasshouse condition, five plants of each line were grown in a 2-L pot filled with commercial potting mixture with a distance of 0.2 m between each pot on 7 May 2020. All trials were conducted in a randomized complete block design with three replicates. To evaluate the anatomical properties of the stems, five stems were selected at random from each DH line at post-anthesis and were cross-sectionally cut at the center of every third internode. All samples were collected from main tillers.

### Morphology Measuring Methods

**Figure 1:**
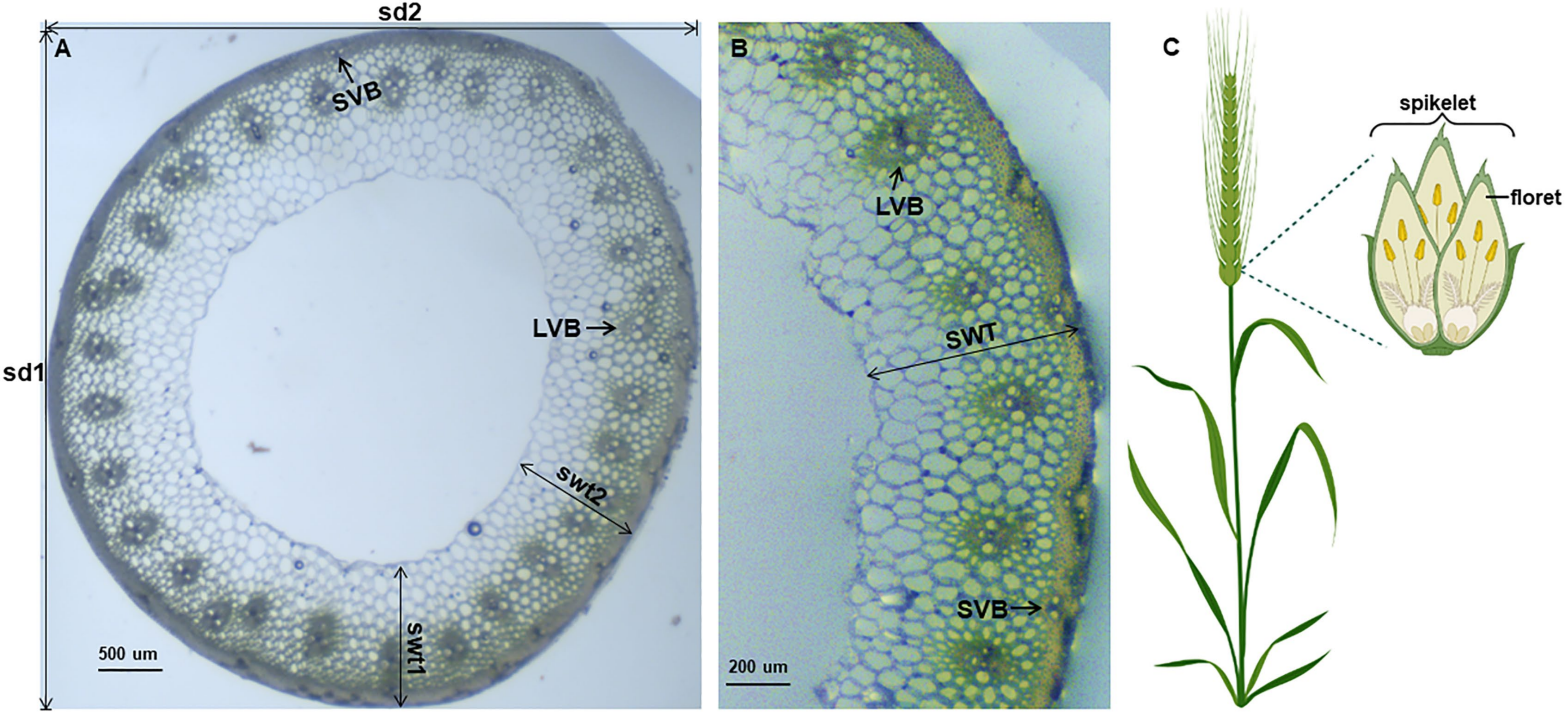
Display of measured wheat characteristics. Anatomical characteristics of wheat stem **(A,B)**. The wheat stems are not that regularly round, so we measured the longest (sd1) and the shortest stem diameter (sd2), and the thickest (swt1) and the thinnest (swt2) stem wall for data collecting. LVB: large vascular bundles; SVB: small vascular bundles; SD: stem diameter; SWT: stem wall thickness. Wheat spikelet architecture **(C)**, created with BioRender.com.

Since wheat stems are not as regularly round, we choose the vernier caliper method to measure stem diameter: the stem diameter (SD) = 1/2 (longest diameter (sd1) + shortest diameter (sd2); Figures 1A,B). A transverse loop (less than 0.1 cm in width) in the middle of the third internode was cut by razor blades, observed under a microscope and pictures were taken. The number of the big vascular bundle (VB) was counted. Stem wall thickness was the average of the thickest (swt1) and the thinnest (swt2) stem wall of each cross-section (Figures 1A,B). At maturity, five panicles were collected from each line to count the number of the spikelet (RN; Figure 1C), and measure the total grain weight (PW, g). Plant height (PH, cm) was measured from soil surface to the top of the spike excluding the awns in the field.

### Statistical Analysis

Data processing for QTL mapping was described in a previous study (Niu et al., 2021). Mean phenotypic values across replicates in each environment and best linear unbiased estimation (BLUE) values across multiple environments were then generated for statistical analysis. The mean values of BLUEs of each lines were used for QTL mapping for measured traits (Supplementary Table S1). Pearson’s correlation coefficients were analyzed using GraphPad Software, San Diego, California USA.^1^ Plots for the distribution of phenotypes were conducted on the data visualization web server “ImageGP” (Chen et al., 2022).

### Genotyping and QTL Mapping

DNA was isolated from young leaves of each line, including the parents, using the CTAB method (Murray and Thompson, 1980). Whole genome diversity array technology (DArT) and single nucleotide polymorphism (SNP) genotyping based on the reference genome of the bread wheat variety Chinese Spring (IWGSC RefSeq v2.0, International Wheat Genome Sequencing Consortium)^2^ assembly were conducted by Diversity Arrays Technology (Canberra, Australia).^3^ The genetic map was constructed in JoinMap 4.0 (Ooijen et al., 2006) using 2,518 polymorphism markers (*χ^2^*-test, *p* < 0.05) with <10% missing data (Supplementary Table S2). The genetic and physical positions of the markers were aligned with the Chinese Spring wheat reference genome assembly (Alaux et al., 2018), the order of the markers on each chromosome in the linkage map was compared with the order of the physical map of each chromosome (Supplementary Figure S1). QTL analysis was conducted with MapQTL 6.0 (Van Ooijen, 2009). Digenic interactions analysis between non-allelic QTL were similar to the previously reported (Fan et al., 2015; Gill et al., 2017). The R package LinkageMapView (Ouellette et al., 2017) was used to visualize the constructed map. MapChart 2.2 (Voorrips, 2002) was used for the plotting linkage groups and QTL locations.

## Results

### Construction of the Genetic Map

The genetic map was generated from 451 high-quality polymorphic SNP markers and 2067 DArT markers covering a total map distance of 4996.1 cM in 21 linkage groups corresponding to the 21 wheat chromosomes, with chromosome sizes ranging from 73.7 cM (4D) to 325.5 cM (2D). On average, each chromosome contained 120 markers, ranging from 19 (4D) to 184 on (5B), and the total marker density was 1.87 cM, ranging from 0.65 cM on 6B to 6.52 cM on 5D (Table 1; Supplementary Tables S1–S3).

**Table 1:** Linkage analysis of molecular markers in Baiqimai/Neixiang 5 DH population.

Linkage group	Number of markers	Physical interval (Mb)	Genetic length (cM)	Avg. inter marker distance (cM)
1A	112	Chr1A_10.4–749.7	228.36	2.04
1B	147	Chr1B_4.6–750.7	218.68	1.49
1D	46	Chr1D_0.8–745.2	175.89	3.82
2A	168	Chr2A_12.5–775.6	252.7	1.5
2B	180	Chr2B_1.5–811.4	260.49	1.45
2D	112	Chr2D_10.7–788.0	325.53	2.91
3A	158	Chr3A_0.9–815.1	276.43	1.75
3B	149	Chr3B_1.4–824.3	232.97	1.56
3D	61	Chr3D_0.9–619.3	255.26	4.18
4A	143	Chr4A_0.2–763.5	185.15	1.29
4B	135	Chr4B_6.7–778.4	180.68	1.34
4D	19	Chr4D_10.5–636.9	73.73	3.88
5A	150	Chr5A_2.1–708.5	294.3	1.96
5B	184	Chr5B_2.6–700.2	262.96	1.43
5D	42	Chr5D_3.3–711.3	273.94	6.52
6A	129	Chr6A_2.0–711.6	212.71	1.65
6B	178	Chr6B_0.7–708.3	114.87	0.65
6D	57	Chr6D_5.1–719.4	173.23	3.04
7A	147	Chr7A_0.5–778.4	312.25	2.12
7B	160	Chr7B_23.4–745.1	171.65	1.07
7D	41	Chr7D_3.1–610.0	214.34	5.23
Total	2,518		4696.11	1.87
Average	119.9		223.62	1.87

**Figure 2:**
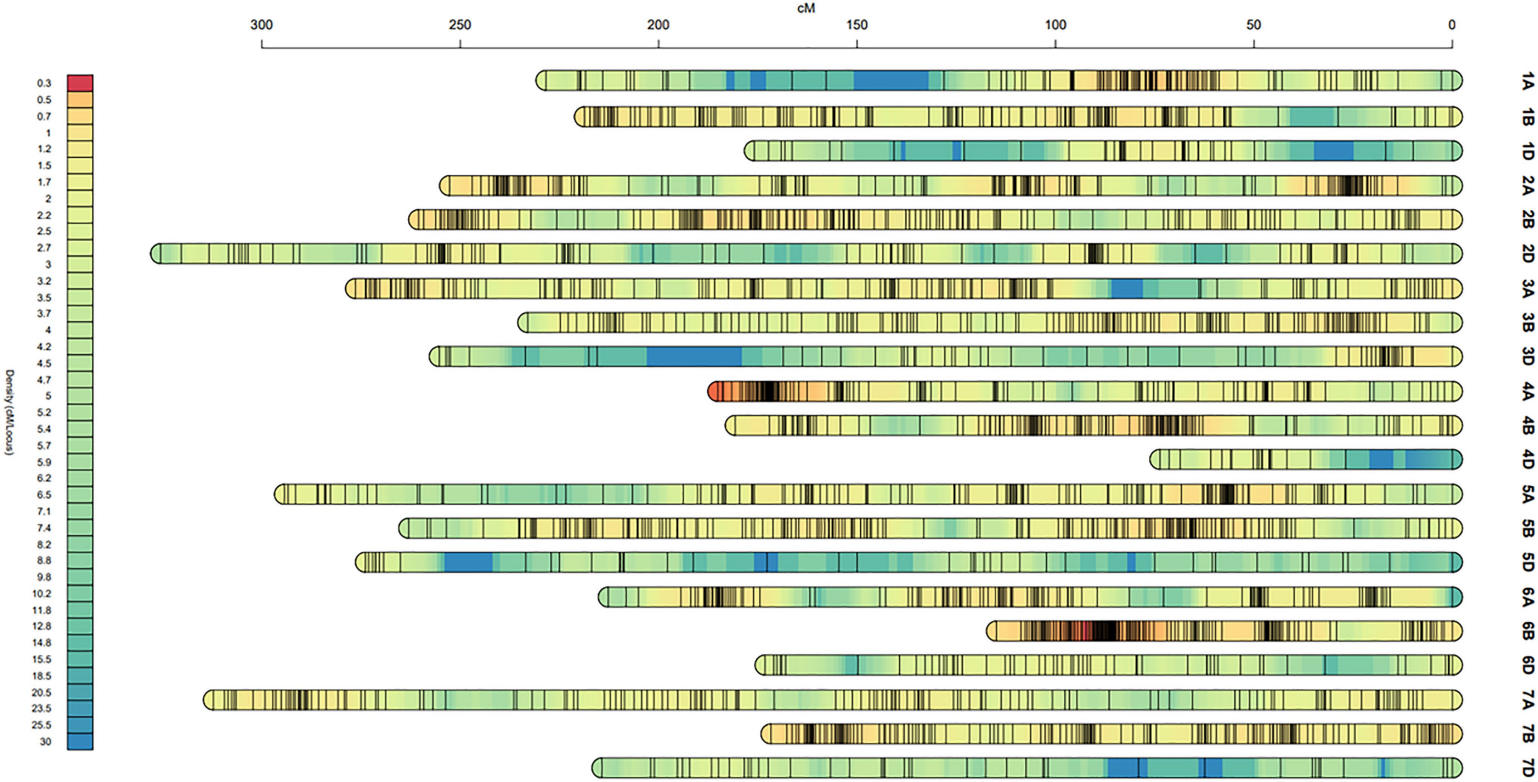
Genetic map constructed from the DH lines from Baiqimai/Neixiang 5.

The marker positions on each chromosome in this map were similar to the published genetic maps (Maccaferri et al., 2015; Wen et al., 2017; Qu et al., 2021). In comparison with genomes A and B, genome D was the shortest and contained much fewer markers and more gaps (Figure 2), suggesting that fewer crossing-over events occurred on the D genome. In general, a high collinearity at the genome-wide level was observed between the genetic and published Chinese Spring consensus map. Lower collinearities were observed in some chromosomal regions due to low marker densities (Supplementary Figure S1), which has also been reported previously (Wingen et al., 2017; Qu et al., 2021). Recombination happened much more frequently in distal chromosomal regions, while recombination near the centromeres tended to be suppressed, consistent with previous studies (Sourdille et al., 2004). The longer map length was due to (1) increased recombination events and map resolution with an higher number of markers and density (Ferreira et al., 2006; Wingen et al., 2017), and (2) differences in chromosomal structure in different mapping populations and application of different ordering algorithms (Ferreira et al., 2006; Qu et al., 2021).

### Phenotypic Variations and Correlations of the DH Lines

**Figure 3:**
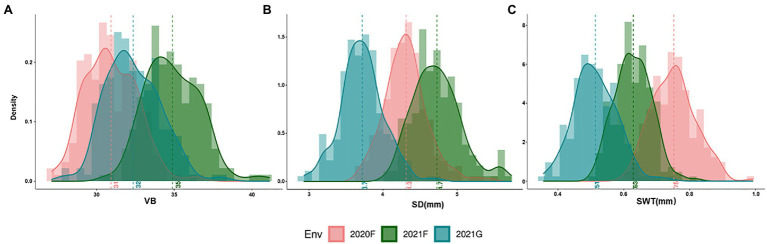
Phenotypic distributions of VB **(A)**, SD **(B)** and SWT **(C)** in three environments. VB: number of big vascular bundle; SD: stem diameter; SWT: stem wall thickness.

**Figure 4:**
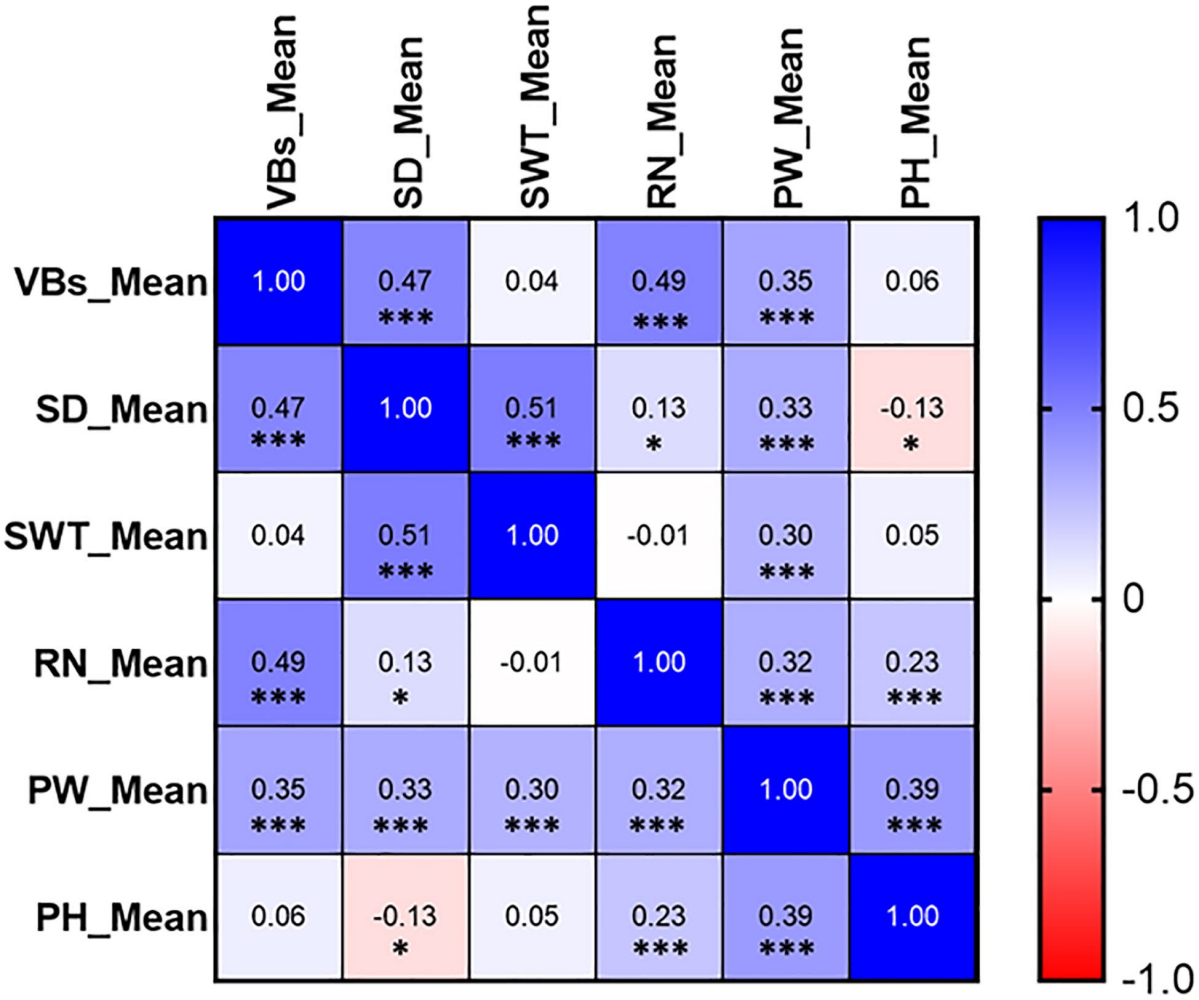
Correlations between stem- and yield-related traits. The number in the middle of the cell is the correlation coefficient; “^*^” and “^***^” refer to significant correlations (*p* < 0.05 and *p* < 0.001). VB: number of big vascular bundle; SD: stem diameter; SWT: stem wall thickness; RN: the number of the spikelet; PW: panicle weight; PH: plant height.

The frequency distribution of each measured trait showed a continuous distribution, with all measured traits in the DH lines across all environments exhibiting significant differences between genotypes (Figure 3; Supplementary Figure S2). VB was significantly positively correlated not only with SD but also with yield-related traits RN and PW, while the correlations with SWT and PH did not reach the level of significance (Figure 4). The correlation coefficients for SD and other traits ranged from 0.13 to 0.51 (*p* < 0.05), and the correlation coefficients for PW with the rest of the measured traits ranged from 0.30 to 0.39 (*p* < 0.001). SWT was significantly associated with PW with a correlation coefficient of 0.30 (*p* < 0.001). In addition, data from the three harvests in 2020 and 2021 showed high correlation coefficients for all measured traits (Supplementary Figure S3). All five traits showed very high heritability ranging from 0.78 for SD to 0.92 for RN across different environments, except PW that had only 0.28 (Table 2; Supplementary Figure S3).

**Table 2:** Variance components and heritability estimates for stem- and yield-related traits.

Trait	V_g_	V_gei_	V_e_	Replication	Environments	*h^2^*
VB	3	0.54	2.97	3	3	0.85
SD	0.07	0.03	0.08	3	3	0.78
SWT	0.06	0.02	0.08	3	3	0.79
PW	1.41	4.73	8.14	3	2	0.28
RN	1.73	0.2	0.83	3	3	0.92

*V_g_: genotype variance; V_e_: residual error variance; V_gei_: genotype by environment interaction variance; h^2^: narrow-sense heritability. VB: number of big vascular bundle; SD: stem diameter; SWT: stem wall thickness; RN: the number of the spikelet; PW: panicle weight; PH: plant height*.

### QTL for Different Traits

**Figure 5:**
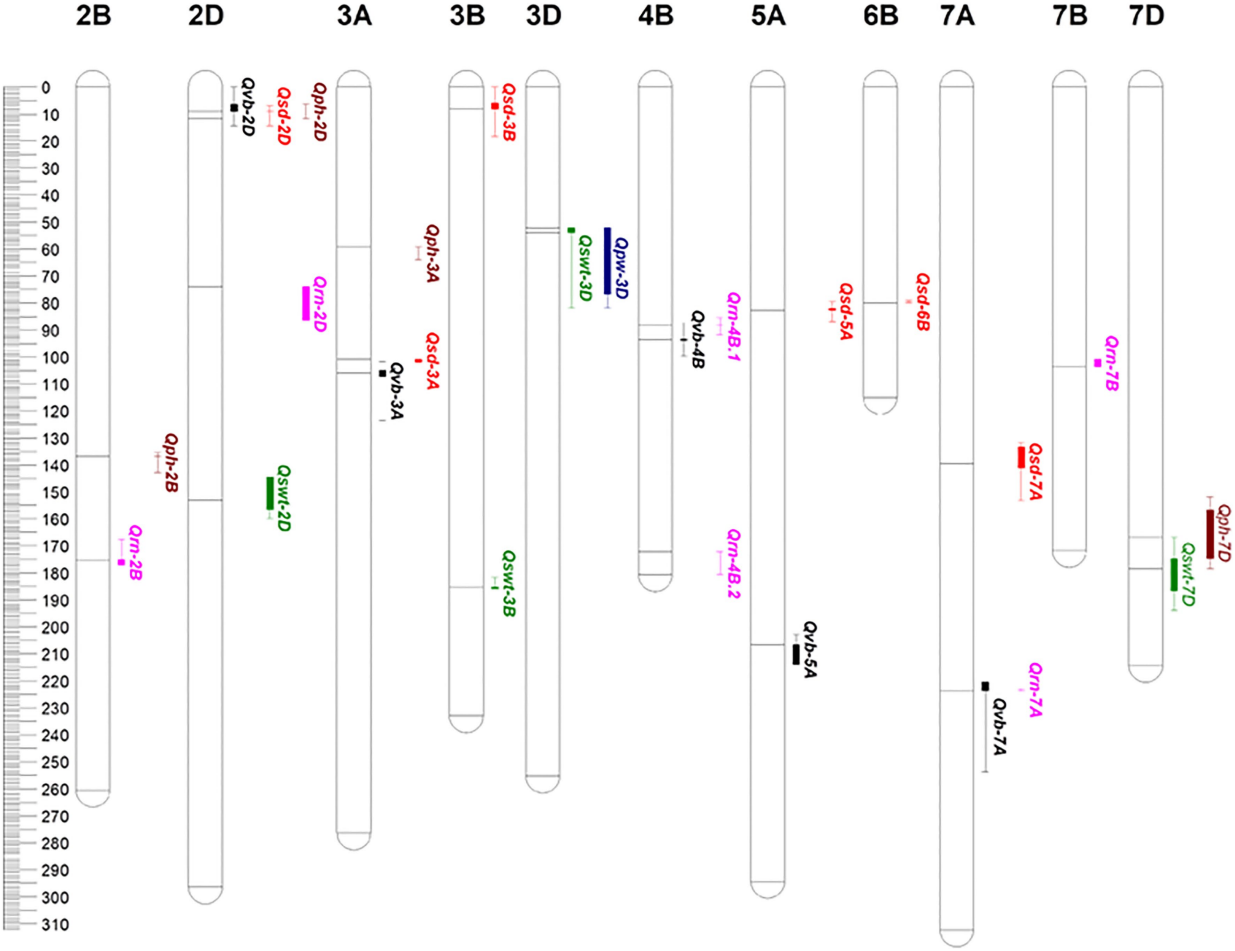
QTL identified for stem- and yield-related traits. The solid line near each QTL within each chromosome indicate the nearest marker of individual QTL, respectively. vb: number of big vascular bundle; sd: stem diameter; swt: stem wall thickness; rn: the number of the spikelet; pw: panicle weight; ph: plant height. Black color for VB; red color for SD; green color for SWT; purple color for RN; blue color for PW; brown color for PH.

Five QTL for VB were identified on chromosomes 2D, 3A, 4B, 5A, and 7A and designated *Qvb-2D*, *Qvb-3A*, *Qvb-4B*, *Qvb-5A*, and *Qvb-7A*, respectively (Figure 5; Table 3). *Qvb-5A* with 5357328 as the nearest marker explained 15.4% of phenotypic variation. The other four QTL explained a total of 24.3% of the phenotypic variation. The closest markers for these four QTL were D1179294-2D21.4, 7487752—0, 1120870-4B43.8, and D1107943-7A1150, respectively. Six QTL for SD were detected on 2D (*Qsd-2D*), 3A (*Qsd-3A*), 3B (*Qsd-3B*), 5A (*Qsd-5A*), 6B (*Qsd-6B*), and 7A (*Qsd-7A*), with the two major ones, *Qsd-3A* and *Qsd-5A* determining 12.6 and 11.5% of the phenotypic variation, respectively. Four QTL for SWT were mapped to chromosomes 2D, 3B, 3D, and 7D, respectively. The major QTL, *Qswt-3B* with the nearest marker of D3942570-2B81.7 determined 13.4% of the phenotypic variation. *Qvb-2D* and *Qsd-2D* were located at the same position (38.09 cM) and *Qvb-3A* overlapped the region of *Qsd-3A* (Figure 5; Table 3), confirming the significant correlation between VB and SD (Figure 4).

**Table 3:** QTL for stem- and yield-related traits.

Traits^a^	QTL	Chromosome	Position (cM)	Nearest marker	2-LOD interval (cM)	LOD score	Percent phenotypic variation explained (*R^2^*, %)^b^	Allele effects^c^	Comparison with reported QTL
VB	*Qvb-2D*	2D	38.086	D1179294–2D21.4	29.06–43.45	4.02	5.7	0.385831	*Rht8/RNHL-D1* (Chai et al., 2022; Xiong et al., 2022)
	*Qvb-3A*	3A	105.88	7487752-0	101.67–123.51	4.66	6.7	−0.417795	
	*Qvb-4B*	4B	93.63	1120870-4B43.8	87.21–99.71	4.6	6.6	−0.423922	
	*Qvb-5A*	5A	206.56	5357328	202.82–214.01	10.06	15.4	−0.634144	
	*Qvb-7A*	7A	223.67	D1107943–7A1150	220.44–253.71	3.71	5.3	0.370098	
SD	*Qsd-2D*	2D	38.09	D1179294–2D21.4	35.99–43.45	6.56	9.4	0.0922892	*Rht8/RNHL-D1* (Chai et al., 2022; Xiong et al., 2022)
	*Qsd-3A*	3A	100.82	D4260815–3A41.9	100.82–102.02	8.55	12.6	−0.105789	*3A* (Berry and Berry, 2015)
	*Qsd-3B*	3B	8.24	3022720-3B9.02	0–18.33	3.44	4.8	−0.0647802	
	*Qsd-5A*	5A	82.71	D1164760–5A51.8	79.52–86.90	7.86	11.5	−0.100992	
	*Qsd-6B*	6B	79.94	D1708171	79.06–79.94	6.28	9	−0.0901461	*6B* (Berry and Berry, 2015)
	*Qsd-7A*	7A	139.45	D1182394–3A16.4	131.81–153.29	3.46	4.8	−0.0646935	
SWT	*Qswt-2D*	2D	182.2	D5332256–2B41.5	173.53–189.01	5.1	8.2	0.08053	
	*Qswt-3B*	3B	185.37	D3942570–2B81.7	181.66–185.94	8.03	13.4	−0.102763	*Qss.msub-3BL* (Oiestad et al., 2017)
	*Qswt-3D*	3D	54.01	D1116044–3D16.7	52.07–81.79	5.14	8.3	−0.0806452	
	*Qswt-7D*	7D	178.44	D4990057–7D97.7	166.78–193.98	3.73	5.9	−0.0686018	*TaSEP3-D1* (Zhang et al., 2021)
PW	*Qpw-3D*	3D	52.07	D1104351–3D16.3	52.07–81.79	3.05	7	−0.653604	
RN	*Qrn-2B*	2B	175.36	D1080886–2B82.9	167.62–177.09	3.17	3.2	0.277751	
	*Qrn-2D*	2D	103.12	D1268308–2D60.6	103.12–115.54	5.37	5.6	−0.337096	
	*Qrn-4B.1*	4B	88.33	D1084202–4B45.30	85.50–91.66	5.18	5.4	−0.3377	
	*Qrn-4B.2*	4B	172.13	D5993254	172.13–180.68	7.33	7.9	0.390091	
	*Qrn-7A*	7A	223.67	D1107943–7A1150	223.05–223.67	21.3	27.3	0.726702	*WAPO1* (Kuzay et al., 2019)
	*Qrn-7B*	7B	103.7	D1243183–5B65	100.78–103.70	5.18	5.4	−0.325627	*WAPO1-7B* (Kuzay et al., 2019)
PH	*Qph-2B*	2B	136.82	D2301818–2B74.1	135.26–142.82	4.95	7.1	−3.24011	
	*Qph-2D*	2D	40.68	D7347063–0	35.46–40.68	9.56	15.8	−4.58189	*Rht8/RNHL-D1* (Chai et al., 2022; Xiong et al., 2022)
	*Qph-3A*	3A	59.26	D4329684	59.26–63.88	5.25	7.6	3.18915	
	*Qph-7D*	7D	166.78	992105	151.95–178.44	4.39	6.3	−2.89355	*TaSEP3-D1* (Zhang et al., 2021)

a *For trait abbreviations, see* Table 2.

b *Percentage of the phenotypic variation explained by the QTL.*

c *Additive effect: positive values mean genotype “a” alleles increased phenotypic values while negative values of the additive effect mean genotype “a” alleles decreased trait scores.*

QTL were also mapped for PW, RN and PH. The major QTL for PH (*Qph-2D*) was overlapped with only one minor QTL for VB (*Qvb-2D*) and one for SD (*Qsd-2D*), *Qph-7D* on chromosome 7D was overlapped with only one minor QTL for SWT (*Qswt-7D*), confirming the less significant correlations between PH and these three stem traits. One major QTL for RN was identified on chromosome 7A (*Qrn-7A*) which is co-located with (*Qrn-7A*). Another minor QTL on chromosome 4B (*Qrn-4B.1*) is co-located with *Qvb-4B*, indicating potential relationship between RN and VB (Figure 4). Only one minor QTL was identified for PW (*Qpw-3D*) due to its low heritability (Table 2) and this QTL was at a similar position to a QTL for SWT on chromosome 3D (*Qswt-3D*; Table 3; Figure 5).

### The Effect of PH on QTL for Stem-Related Traits

Plant height is one of the most important features of plants’ architecture affecting plant lodging resistance under harsh environmental conditions (Berry and Berry, 2015). However, as shown in Figure 4, only SD exhibited a negatively close association with PH (−0.13, *p* < 0.05), and the major QTL for stem-related traits were totally different with that for PH. To further confirm the relationships between PH and those stem-related traits in wheat, QTL analyses were performed using PH as a covariate. Of the five QTL for VB, all QTL showed increased *R^2^* with the percentage of the phenotypic variation determined by the major QTL *Qvb-5A* being from 15.4 to 18.5% (Tables 3, 4), while the remaining four minor QTL for the trait showed decreased *R^2^* (Tables 3, 4), when using PH as a covariate. In contrast, when PH was used as a covariate, *Qsd-2D* and *Qsd-3B* became insignificant, *Qsd-3A* showed a slight reduction in *R^2^*, *Qsd-5A* and *Qsd-7A* showed a slight increase in *R^2^*, and *Qsd-6B* remained the same (Table 4). The *R^2^* of *Qswt-2D* for SWT showed a slight increase (from 8.2 to 10.2%), and the *R^2^* of all other QTL for SWT had a slight decrease when using PH as a covariate (Table 4). In general, PH was independent of VB, although *Qvb-2D* and *Qph-2D* were co-located at the same position on 2D (Table 3; Figure 5).

**Table 4:** Changes in the significance and percentage variation determined by the QTL after PH or SWT/VB were used as covariates.

QTL	Chromosome	Covariate	Position	Nearest marker	LOD	*R^2^*
*Qvb-2D*	2D	PH	38.09	D1179294-2D21.4	4.96	5.3
*Qvb-3A*	3A	PH	105.88	7487752--0	5.9	5.9
*Qvb-4B*	4B	PH	93.63	1120870-4B43.8	5.76	6.2
*Qvb-5A*	5A	PH	206.56	5357328-	16.3	18.5
*Qvb-7A*	7A	PH	223.67	D1107943-7A1150	5.16	5.2
*Qsd-2D*	2D	PH	ns	ns	ns	ns
*Qsd-3A*	3A	PH	100.82	D4260815-3A41.9	8.35	11.9
*Qsd-3B*	3B	PH	ns	ns	ns	ns
*Qsd-5A*	5A	PH	82.71	D1164760-5A51.8	8.52	12.2
*Qsd-6B*	6B	PH	79.94	D1708171	6.23	9
*Qsd-7A*	7A	PH	139.4	D1182394-3A16.4	4.53	6.2
*Qswt-2D*	2D	PH	182.20	D5332256-2B41.5	6.74	10.2
*Qswt-3B*	3B	PH	185.37	D3942570-2B81.7	5.85	8.8
*Qswt-3D*	3D	PH	54.01	D1116044-3D16.7	3.71	5.4
*Qswt-7D*	7D	PH	178.44	D4990057-7D97.7	3.42	5.2
*Qsd-2D*	2D	SWT	36.49	1242814-2D20.9-0	9.57	7.3
*Qsd-3A*	3A	SWT	101.67	2254081-3A49	4.81	5.9
*Qsd-3B*	3B	SWT	ns	ns	ns	ns
*Qsd-5A*	5A	SWT	82.71	D1164760-5A51.8	6.1	7.6
*Qsd-6B*	6B	SWT	ns	ns	ns	ns
*Qsd-7A*	7A	SWT	ns	ns	ns	ns
*Qpw-3D*	3D	VB	52.07	D1104351-3D16.3	3.68	7.3
*Qrn-2B*	2B	VB	ns	ns	ns	ns
*Qrn-2D*	2D	VB	103.12	D1268308-2D60.6	3.27	3.2
*Qrn-4B.1*	4B	VB	ns	ns	ns	ns
*Qrn-4B.2*	4B	VB	ns	ns	ns	ns
*Qrn-7A*	7A	VB	223.67	D1107943-7A1150	18.07	20.9
*Qrn-7B*	7B	VB	ns	ns	ns	ns

*ns means not significant; trait abbreviations see* Table 2.

### The Effect of VB on QTL for Yield-Related Traits

VB was also strongly correlated with RN and PW in the current study (Figure 4). No significant changes to the QTL for PW when using VB as a covariate. However, four out of six QTL for RN became insignificant when using VB as a covariate and the *R^2^* of the major QTL for RN on 7A (*Qrn-7A*) showed a slight decrease from 27.3 to 20.9% (Table 4).

## Discussion

Several genes have been reported to affect VB formation in *Arapidopsis* and rice (Hardtke and Berleth, 1998; McConnell et al., 2001; Zhong and Ye, 2004; Qi et al., 2008; Terao et al., 2010; Fujita et al., 2013; Du and Wang, 2015; Fei et al., 2019). However, there have been no reports on QTL for the number of VB in wheat stem with most studies being concentrated on the regions close to the neck of the spike (Sang et al., 2010). Here, we have found that the VB number in the third internode showed the greatest variation and several QTL were responsible for VB number. A major novel QTL affecting VB the third internode was identified on chromosome 5A [*Qvb-5A* (206.56–214.01 cM, 612.16–641.76 Mb); Table 3; Figure 5]. According to the annotation database,^4^ *TraesCS5A01G445800* (625,908,587–625,908,859 bp) coding for an auxin efflux carrier family protein is supposed to participate in all aspects of vascular differentiation thus could be one of the candidate genes for *Qvb-5A*. One of the theories to explain how auxin regulates the formation of regular patterns of vascular tissue distribution in plants is the canalization of auxin flow (Biedroń and Banasiak, 2018). *Narrow leaf1* (*nal1*) in rice, which is abundantly expressed in vascular tissues, affects polar auxin transport and vascular patterns in rice plants (Qi et al., 2008). Several auxin efflux carrier family proteins have been reported in *Arabidopsis thaliana*, which were involved in polar auxin transport and accumulation and the formation of vascular tissues (Forestan and Varotto, 2012).

Among all other minor QTL, the *Qvb-2D* on chromosome 2D was located at a similar position to the major wheat semi-dwarfing gene *Rht8*/*RNHL-D1* [*TraesCSU02G024900* (CS RefSeq v1.0, 24 Mb); Chai et al., 2022; Xiong et al., 2022]. The GA-sensitive *Rht8* reduces plant height without scarifying coleoptile length (Chai et al., 2022), and the combinations of *Rht8* and *Rht4* (had moderate effects on plant height) could reduce plant height to a desirable level, and improve yield-related traits in the rainfed cultivation (Yingying et al., 2018). However, our results showed that the QTL for VB was independent on the QTL for plant height with no significant correlation between VB and PH. Hence, validation and/or fine mapping of those new QTL is necessary for finding reliable validated markers to be utilized in marker-assisted selection (MAS) or genomic selection (GS) for stem strength and high yield.

**Figure 6:**
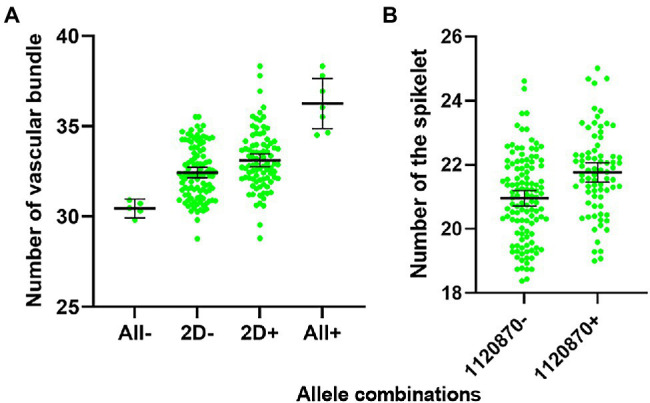
Allele effects of VB alleles on the number of big vascular bundle **(A)**, and the number of the spikelet **(B)**. ALL-stands for all alleles for VB were absent; 2D-stands for allele on Chromosome 2D (*Qvb-2D*) was absent; 2D+ stands for allele on Chromosome 2D (*Qvb-2D*) was present; ALL+ stands for all alleles for VB were present. 1120870 is the nearest marker for *Qvb-4B*, 1120870- means 1120870 was absent; 1120870+ means 1120870 was present. Error bars in the figures indicate 95% confidence interval.

The combination of the positive VB alleles detected in our study significantly increased the number of vascular bundles from 30 to 31 (All−) to 35–38 (All+; Figure 6A). The reported QTL/gene on chromosome 2D only slightly improved the number of vascular bundles with no significant difference between the presence (2D+) and absence (2D−) of 2D allele. Therefore, the new discovered QTL for VB present great potential in improving stem strength by pyramiding of major QTL for large number of vascular bundles. The pattern of mechanical development of winter wheat can maximize its reproductive success (Crook et al., 1994). In this study, VB showed a significant correlation with RN which was confirmed by QTL analysis for RN using VB as a covariate (Table 4). The increasing allele represented by the closest marker 1120870 for VB 4B QTL also showed a significant increase in RN (Figure 6B). The major QTL on 5A (*Qvb-5A*) showed slight but positive impacts on RN.

Increasing stem strength with a minimal investment in biomass can be achieved by increasing internode diameter and material strength rather than internode wall width (Berry et al., 2007; Piñera-Chavez et al., 2021). Among the six QTL for SD, *Qsd-2D* was at a similar position to *Qvb-2D* and the major QTL for PH. *Qsd-3A* and *Qsd-6B* have been reported earlier (Berry and Berry, 2015). *Qsd-3A* for SD (100.82–102.02 cM) is located at a similar position to *Qvb-3A* for VB (101.67–123.51 cM) with increasing alleles of both QTL being from the parent “Neixiang 5” (Table 3; Figure 5), indicating potential pleiotropic effects on overall stem strength or tightly linked genes in this QTL region. The other major QTL (*Qsd-5A*) was a novel one from this population (Table 3; Figure 5). Candidate genes, such as *TraesCS5A01G239400* (Bri1 kinase inhibitor 1), and two cellulose synthase genes (*TraesCS5A01G253100* and *TraesCS5A01G253200*) have great potential on regulating cell expansion and elongation (Hyles et al., 2017; Oh et al., 2020).

SWT showed no significant correlation with VB, but it was positively associated with SD (0.51, *p* < 0.001, Figure 4). To further investigate the relationship between SWT and SD, we performed the QTL analysis for SD using SWT as a covariate. *Qsd-3B*, *Qsd-6B*, and *Qsd-7A* became insignificant, and the *R^2^* of the two major QTL (*Qsd-3A* and *Qsd-5A*) for SD reduced significantly from 12.6 to 5.9 and 11.5 to 7.6, respectively (Table 4). The major QTL for SWT (*Qswt-3B*) was located on the long arm of chromosome 3B with a QTL region of between 181.66 and 185.94 cM (771.56–775.09 Mb), which coincides with *Qss.msub-3BL* (761,188,585–773,049,079 bp) for stem solidness (Sherman et al., 2015; Oiestad et al., 2017). No candidate genes responsible for *Qss.msub-3BL* were identified. *Qswt-3D* has not been reported before. A gene encoding Rho of Plants (ROP) proteins (*TraesCS3D01G057400*, 24,090,998–24,095,444 bp), also known as RACs, is the most likely candidate gene for this QTL. ROPs are involved in the regulation of abscisic acid (ABA) and auxin signaling and transport (Wu et al., 2011; Liao et al., 2017), and also regulate cell polarization and secondary cell wall development in the xylem of plants (Bloch and Yalovsky, 2013; Oda and Fukuda, 2014; Feiguelman et al., 2018). The QTL interval of *Qswt-7D* (166.781–193.984 cM, 177.88–413.11 Mb) overlapped with *Qph-7D* (151.948–178.44 cM, 135.40–384.45 Mb) where a MADS-box gene *TaSEP3-D1* (*TraesCS7D02G261600*, Refv1.0, chr7D:237.609–237.619 Mb) that regulates both heading date and plant height development (Zhang et al., 2021) is located.

## Conclusion

In conclusion, QTL were identified for the stem traits of the third internode, which showed the greatest variation and is probably the most important part linked to lodging resistance. The number of VB in the third internode showed significant correlation with the number spikelets. The combination of positive alleles of the QTL for VB can increase VB by more than 15%. QTL were also identified from stem wall thickness and diameter. Most of the QTL for these traits showed no significant correlation with plant height. The results offer great opportunities for improving stem lodging resistance and improving yield components with less effect on PH.

## Data Availability Statement

The original contributions presented in the study are included in the article/Supplementary Materials; further inquiries can be directed to the corresponding author.

## Author Contributions

MZ acquired the funding and participated in supervision. YN and TC conducted the field trials, data collection, and data analysis and carried out data visualization. CG collected the data. YN wrote the draft. MZ and YN wrote, reviewed, and edited the draft. CZ participated in supervision. All authors contributed to the article and approved the submitted version.

## Funding

This project is funded by the Grains Research and Development Corporation (GRDC) of Australia. The funder had the following involvement with the study: Identification of QTL for stem traits in wheat (*T. aestivum* L.).

## Conflict of Interest

The authors declare that the research was conducted in the absence of any commercial or financial relationships that could be construed as a potential conflict of interest.

## Publisher’s Note

All claims expressed in this article are solely those of the authors and do not necessarily represent those of their affiliated organizations, or those of the publisher, the editors and the reviewers. Any product that may be evaluated in this article, or claim that may be made by its manufacturer, is not guaranteed or endorsed by the publisher.

## Footnotes

^1^ www.graphpad.com

^2^ https://urgi.versailles.inra.fr/download/iwgsc/IWGSC_RefSeq_Assemblies/v2.0/

^3^ https://www.diversityarrays.com

^4^ https://webblast.ipk-gatersleben.de/downloads/wheat/

## Supplementary Material

Supplementary Figure S1 Collinearity of marker orders between the genetic and reported consensus maps.

Supplementary Figure S2 Phenotypic distributions of two yield-related traits and PH in different environments. RN: the number of the spikelet; PW: panicle weight; PH: plant height.

Supplementary Figure S3 Correlations between lodging-related traits and yield-related traits across different environments. The number in the middle of the cell is the correlation coefficient; “^*^,” “^**^,” and “^***^” refer to significant correlations (*p* < 0.05, *p* < 0.01, and *p* < 0.001). VB: the number of big vascular bundle; SD: stem diameter; SWT: stem wall thickness; RN: the number of the spikelet; PW: panicle weight; PH: plant height.

Supplementary Table S1 Phenotypic data of wheat DH population from Baiqimai and Neixiang 5.

Supplementary Table S2 Genotypic data of wheat DH population from Baiqimai and Neixiang 5.

Supplementary Table S3 Map positions of different SNP and DArT markers.

## Abbreviations

BLUE: Best linear unbiased estimation
DArT: Diversity array technology
DH: Doubled haploid
GS: Genomic selection
MAS: Marker-assisted selection
PH: Plant height
PW: Total grain weight
QTL: Quantitative trait loci
RN: Number of the spikelet
ROP: Rho of Plants
SD: Stem diameter
SNP: Single nucleotide polymorphism
SWT: Stem wall thickness
VB: Number of big vascular bundle
*WAPO1*: WHEAT ORTHOLOG OF APO1
