# Supplementary figures and images for: Identification of QTL for Stem Traits in Wheat (Triticum aestivum L.)
Source: Front Plant Sci. 2022 Jul 14;13:962253. doi: 10.3389/fpls.2022.962253 (PMC9330363; doi:10.3389/fpls.2022.962253)

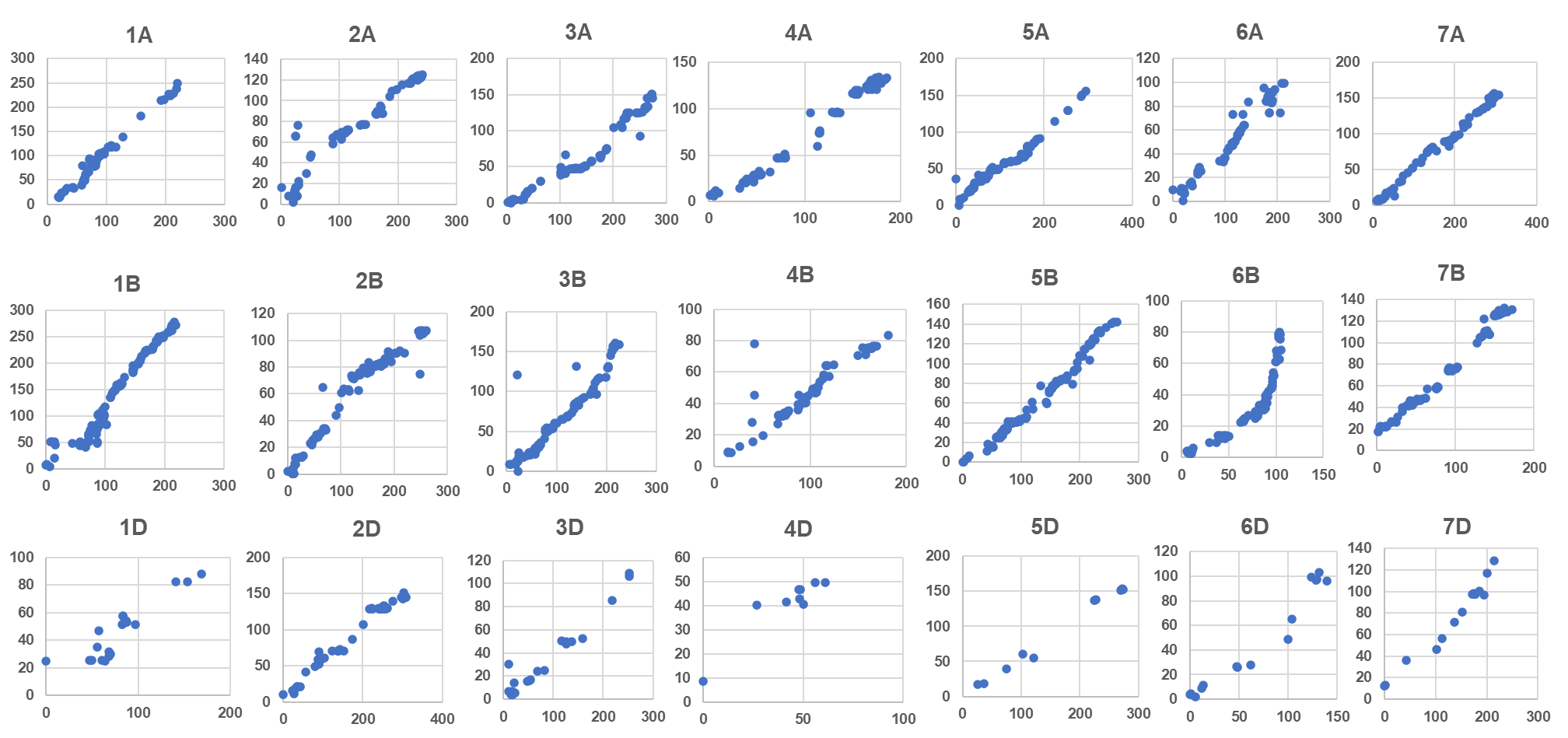
**Fig. S1**


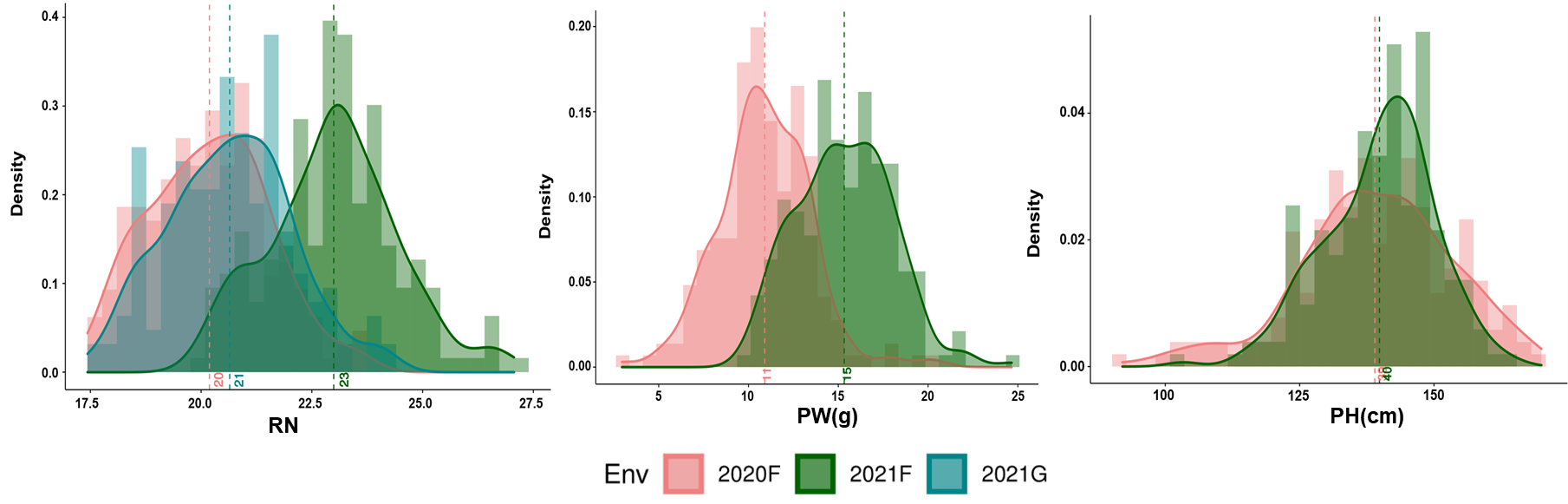


**Fig. S2**


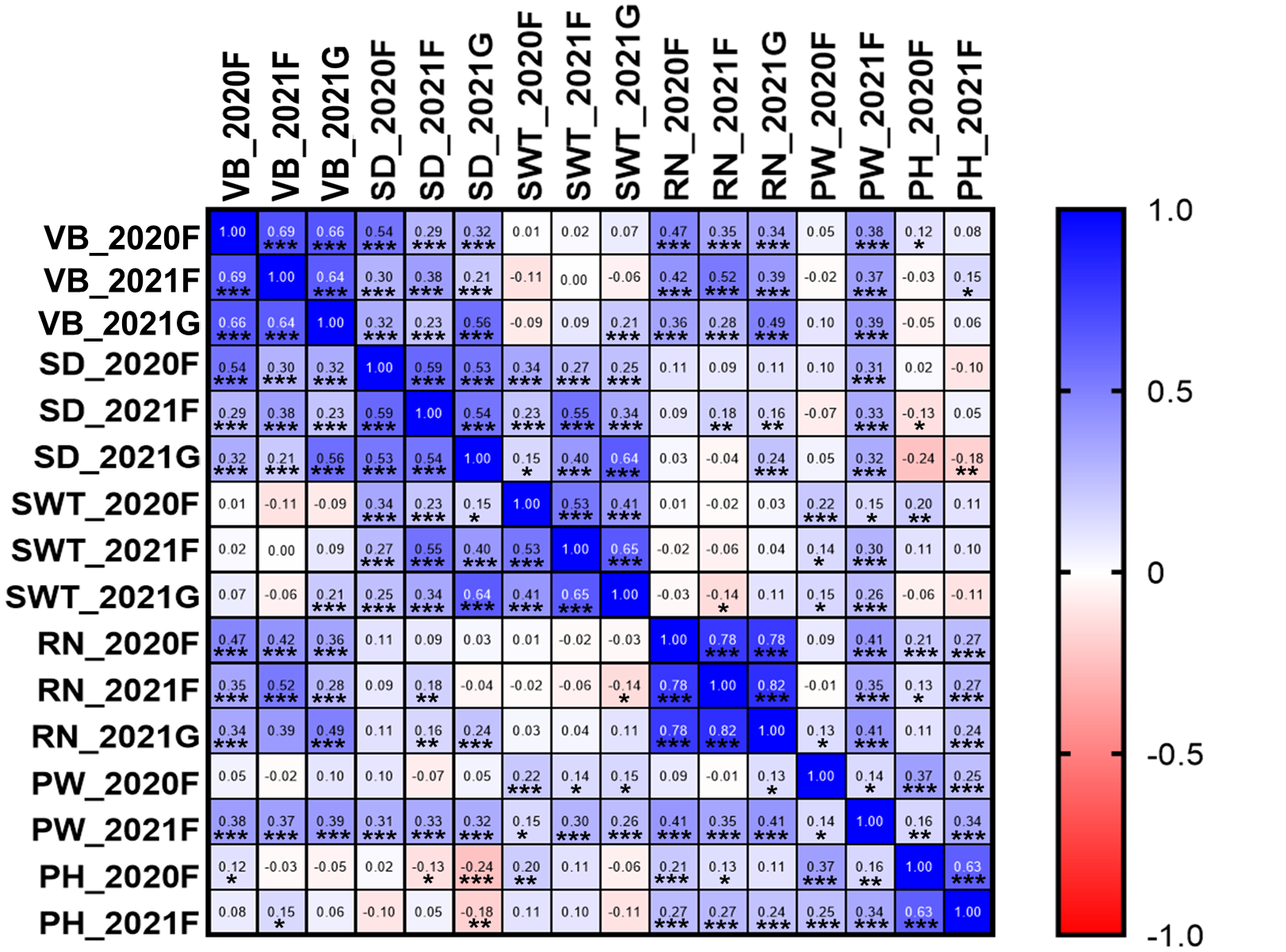


**Fig. S3**

Supplement: Supplementary Figure S1 — Collinearity of marker orders between the genetic and reported consensus maps. [file Data_Sheet_1.docx]
